# What could be the reasons for not losing weight even after following a weight loss program?

**DOI:** 10.1186/s41043-024-00516-4

**Published:** 2024-03-02

**Authors:** Jyoti Dabas, S. Shunmukha Priya, Akshay Alawani, Praveen Budhrani

**Affiliations:** Institute of Nutrition and Fitness Sciences, Platinum Square, 4th floor, Office, 403, Opp. WNS, Sakore Nagar, Viman Nagar, Pune, Maharashtra 411014 India

**Keywords:** Obesity, Lifestyle, Weight loss, Basal metabolism, Hypocaloric diets, Resting metabolic rate, Genetics, Interindividual variability

## Abstract

**Introduction:**

Approximately four million people worldwide die annually because of obesity. Weight loss is commonly recommended as a first-line therapy in overweight and obese patients. Although many individuals attempt to lose weight, not everyone achieves optimal success. Few studies point out that weight loss eventually slows down, stagnates or reverses in 85% of the cases.

**Research question:**

What could be the reasons for not losing weight even after following a weight loss program?

**Methods:**

A scoping review of the literature was performed using weight loss-related search terms such as ‘Obesity,’ ‘Overweight,’ ‘Lifestyle,’ ‘weight loss,’ ‘Basal Metabolism,’ ‘physical activity,’ ‘adherence,’ ‘energy balance,’ ‘Sleep’ and ‘adaptations. The search involved reference tracking and database and web searches (PUBMED, Science Direct, Elsevier, Web of Science and Google Scholar). Original articles and review papers on weight loss involving human participants and adults aged > 18 years were selected. Approximately 231 articles were reviewed, and 185 were included based on the inclusion criteria.

**Design:**

Scoping review.

**Results:**

In this review, the factors associated with not losing weight have broadly been divided into five categories. Studies highlighting each subfactor were critically reviewed and discussed. A wide degree of interindividual variability in weight loss is common in studies even after controlling for variables such as adherence, sex, physical activity and baseline weight. In addition to these variables, variations in factors such as previous weight loss attempts, sleep habits, meal timings and medications can play a crucial role in upregulating or downregulating the association between energy deficit and weight loss results.

**Conclusion:**

This review identifies and clarifies the role of several factors that may hinder weight loss after the exploration of existing evidence. Judging the effectiveness of respective lifestyle interventions by simply observing the ‘general behavior of the groups’ is not always applicable in clinical practice. Each individual must be monitored and advised as per their requirements and challenges.

## Introduction

The World Health Organization (WHO) describes overweight and obesity as an abnormal condition that impairs health causing four million deaths annually [1]. Across the globe, the prevalence of overweight and obesity between 1980 and 2013 increased by 27.5% among adults [2]. Since 1980, a third of the global population has been determined to be obese or overweight [3]. The exact reasons for obesity remain unknown, but there is a complex link between biological, psychosocial and behavioral factors [4]. Further, genetic disposition to obesity [5], unhealthy diet including highly processed foods [9], sedentary lifestyle, disturbed circadian rhythm [6, 7], underlying medical conditions and certain medications [8] all add to the global obesity pandemic [10, 11]. Complexity arises in identifying the real reasons for not losing weight because most of these factors are interlinked, forming reinforcing loops leading to the struggles people have with trying to lose weight.

Numerous studies recommend that a modest weight loss of 5 to 10% significantly improves parameters such as blood glucose levels, blood pressure, inflammatory markers and lipid profile among human participants [12–14]. Hence, to mitigate the risk of developing related metabolic ailments or alleviate existing chronic conditions, individuals with obesity are often advised to reduce weight by expending more energy than they consume (energy deficit) [15]. However simple this advice might seem; it does not seem to work for many [16].

Studies on the ‘prevalence of not losing weight’ are insufficient and authors did not come across any such studies that point to weight loss failures or not losing weight prevalence, instead many RCTs and lifestyle interventional studies highlight the results in terms of successful weight loss. Research has shown that only ≈20% of overweight individuals are successful at long-term weight loss when defined as losing at least 10% of initial body weight and maintaining the loss for at least one year [17].

The internal factors resulting from the complex pathophysiology of obesity such as hormonal imbalance, nutritional and metabolic factors counter the efforts one exerts to lose weight [18]. Additional factors that create interindividual variability in weight loss studies, that are rarely accounted for, include previous weight loss attempts, sleep habits, meal timings and medications which can all play a crucial role in upregulating or downregulating metabolism and weight loss [19, 20].

It follows the effectiveness of respective lifestyle interventions by observing the ‘general behavior of the groups’ is not always applicable in clinical practice where each patient must be advised as per individual requirements and challenges.

In this review, the authors aim to discuss common factors that may hinder weight loss efforts in a lifestyle intervention in the context of clinical applications. Before proceeding, let us understand how *“not losing weight*” is defined. There is no standard definition for not losing weight. Lenoir et al. [21] defined ‘*not losing weight*’ individuals as subjects who did not lose 10% of their initial weight. Other terms used in the article are: ‘*weight loss barrier, weight loss hindrance*,’ or exploring reasons behind why an individual may lose weight at a relatively slower pace is clinically important.

The other objectives include deriving clinically significant interpretations from reviewed evidence and proposing recommendations for future research.

In light of the above observations, the present study has been framed with the research question—What could be the reasons for not losing weight even after following a weight loss program?

## Materials and methods

### Review design

This is a *scoping review*. As a scoping review provides an evaluation of the type and amount of research available on a topic and the potential knowledge gaps, through this article, the authors are addressing the research question ‘reasons for not losing weight despite being on a weight loss program.’

### Search strategy

This research is evidence-based work involving a systematic literature review that utilizes various methods, including reference tracking, database and web searches (PUBMED, Science Direct, Elsevier, Web of Science and Google Scholar), hand-searching of websites and conference proceedings. Due to a lack of results for terms directly related to the study ('unsuccessful weight loss' or 'not losing weight'), the authors expanded the search using terms discovered during initial web searches and their professional experience, such as 'metabolism,' 'lifestyle considerations,' and 'sleep.' The relevant terms were then extracted, edited, grouped and presented using a qualitative meta-summary approach. Key terms that produced significant and relevant findings include 'Obesity,' 'Overweight,' 'Lifestyle,' 'Weight loss,' 'Basal metabolism,' 'physical activity,' 'adherence,' 'energy balance,' 'Sleep,' 'circadian rhythm,' 'adaptations' and 'resting metabolic rate.' These keywords were generated based on the article's relevance.

### Article selection criteria

**Fig. 1:**
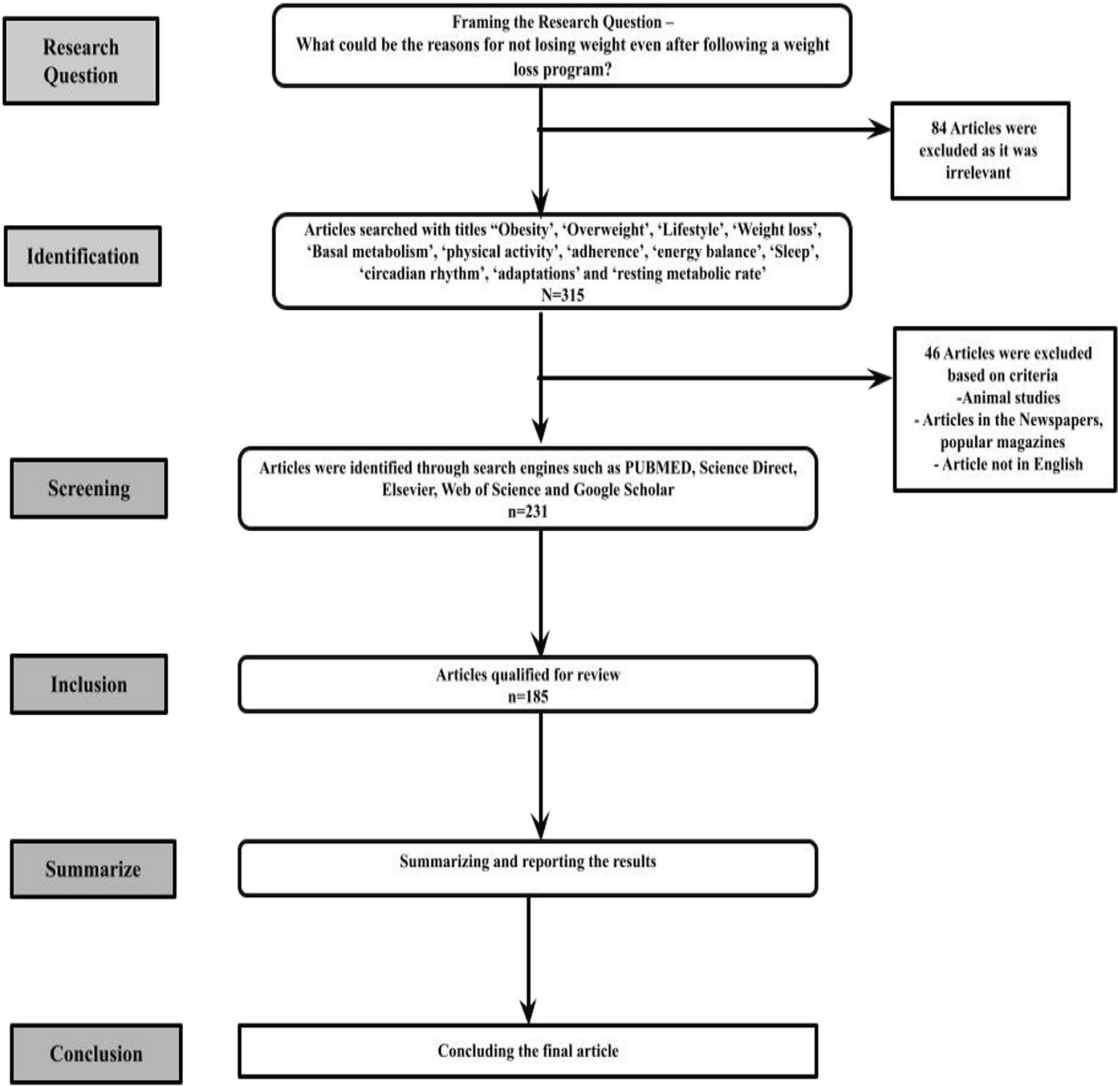
Shows the selection of articles for the study

The selection of the articles for the scoping review was conducted based on the modified method of the Six-Stage Methodological Framework for Scoping Review, adapted from Arksey and O’Malley [22] and Levac et al*.* [23]. Only weight loss original articles/review papers on weight loss studies involving human participants aged > 18 years were considered. Approximately 231 articles were reviewed and 185 articles were included based on the inclusion criteria. Articles in newspapers, magazines and animal studies were excluded. The eligible studies were individually identified by all authors based on the titles, abstracts, full text and references. Also, the duplicate entries were removed. Figure 1 shows flowchart for the selection of articles for the study using the modified method of the Six-Stage Methodological Framework for Scoping Review.

### Study design

The study designs adopted for the paper were original and review articles including randomized controlled trials, cross-sectional studies and case–control studies.

### Study process

The study, initiated in August 2021, involved all authors contributing equally to screening relevant articles for this review. Initially, authors documented observations on reasons for not losing weight. Weekly meetings were held to discuss and organize the first draft, with the second and third authors responsible for the second draft. All authors reviewed each draft in multiple meetings to reach the final article. The second author handled reference checking, and the first author conducted the final review. Manuscript revision was performed by the second author and reviewed by the first author.

### Data extraction

All authors equally contributed to the data extraction process as discussed in the previous section. From the included articles, relevant data were identified and summarized [185]. Data were gathered to answer the research question.

### Ethics approval and consent to participate

For this review paper, the authors have not taken an ethics approval. This is a review paper of already published papers and clinical trials with ethics approval.

## Results

**Fig. 2:**
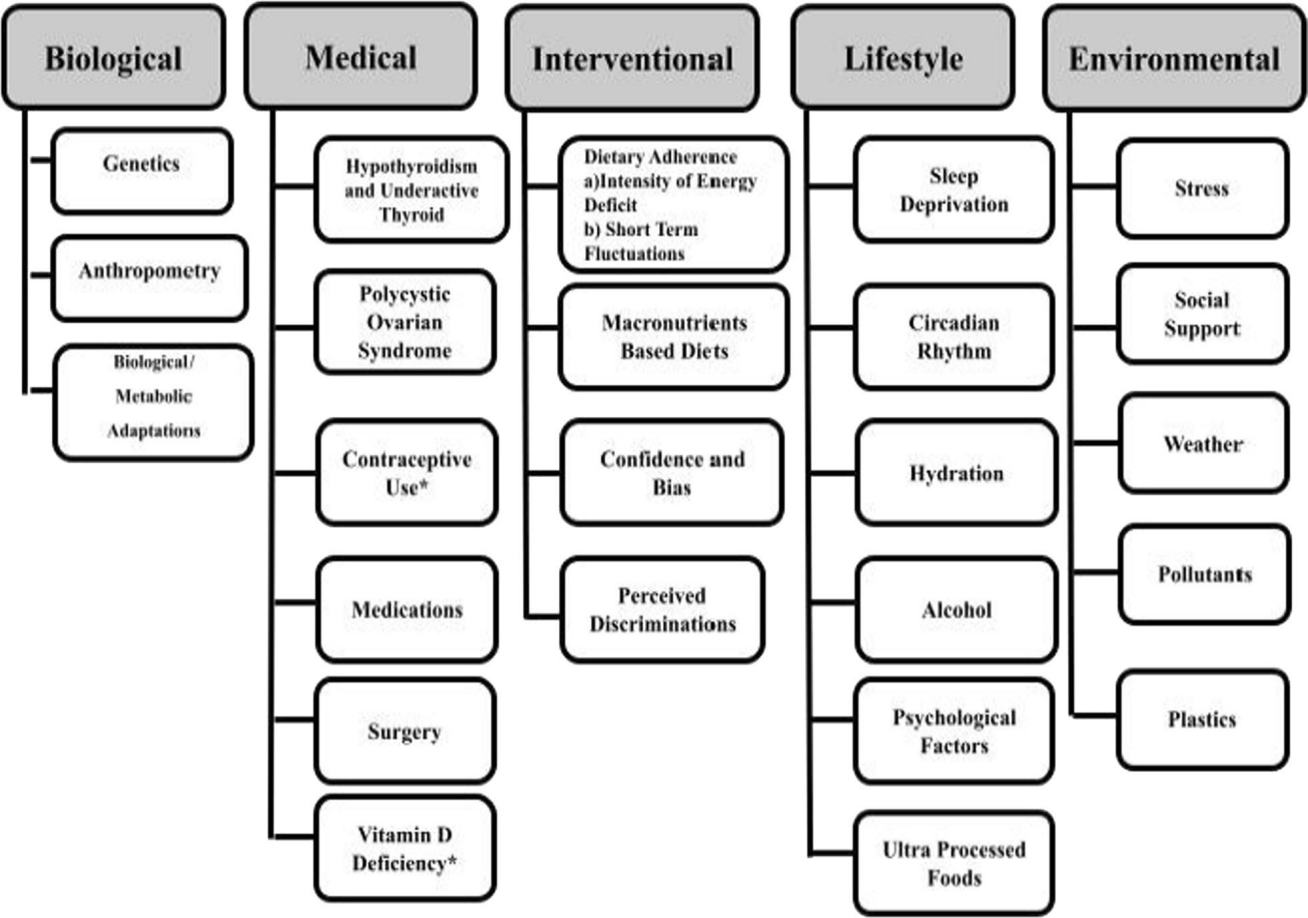
The factors associated with not losing weight

Many factors are associated with not losing weight. In this review, authors have critically evaluated these factors and have categorized the likely common factors hindering weight loss. The possible factors associated with not losing weight have broadly been divided into five categories based on their origin: biological, medical, lifestyle, intervention and environmental. Figure 2 depicts the factors associated with not losing weight among the participants/clients/individuals while following a weight loss program.

I. Biological

The reviewed biological factors hindering weight loss are genetics, anthropometry and metabolic adaptations.

1. Genetics

The genetic component significantly contributes to obesity development, estimated with a heritability of 40–70% [24]. This led researchers to explore genetic variations' role in weight loss as a response to energy deficit. Heritability analysis of weight loss through behavioral intervention in 1080 obese patients claimed 49% phenotypic variance in dietary restriction-induced weight loss is attributed to single nucleotide polymorphisms, with rs679482 (sarcoglycan γ gene), reaching genome-wide significance [25]. Other genes like FTO, MC4R, PPARG and MTIF3 may influence energy expenditure and/or intake affecting weight loss outcomes [26]. The role of genetics in response to energy restriction is complex, and the variation in weight loss can likely be credited to the collective effect of different loci showing small individual effects.

Genetic factors contribute to varied weight loss responses [27]. In the tightly controlled study, individuals who showed a smaller reduction in energy expenditure during fasting and a larger spike in the same during overfeeding predicted more weight loss over six weeks of energy restriction after accounting for other variables like age, sex, race, deficit accumulation and baseline weight (‘thrifty’ phenotype), compared to their counterparts who showed opposite adaptations (‘spendthrift’ phenotype) [28]. Such genetic factors make weight loss more challenging for specific individuals.

2. Anthropometry

In anthropology, factors like height, weight, body composition, size and BMI are considered. Taller or heavier individuals with more fat-free mass typically exhibit a higher basal metabolic rate, potentially facilitating quicker weight loss with similar calorie intake [29–31]. Conversely, shorter and lighter individuals may find weight loss more challenging. However, large sample size studies are lacking in this area. Several studies have identified that those with a BMI > 30 kg/m^2^ struggle with recommended physical activity, whereas those with a BMI of 25 kg/m^2^ perform better [32–37]. Concluding for these trials, high adiposity linked to low physical activity affects weight loss outcomes.

A retrospective study [21] involving 14,256 patients revealed that weight loss success depends on follow-up frequency, initial BMI and initial weight loss. Fewer follow-ups, lower initial BMI and minimal initial weight loss failed to maintain the lost weight beyond a year. Body size, influenced by fat-free mass, plays a vital role in weight loss outcomes. Further, initial success is crucial for eventual success in weight loss efforts.

3. Biological/metabolic adaptations

In response to an energy deficit, resting metabolic rate can notably drop within days, disproportionately to body mass reduction [38, 39]. Termed metabolic adaptation, it is a survival mechanism after significant weight loss. A study on perimenopausal women linked larger metabolic adaptation to prolonged time in achieving weight loss goals, suggesting strategies to decrease it may expedite weight loss [40]. Metabolic adaptation partly explains slower weight loss in later stages of energy deficit, addressed in Hall et al., mathematical model [41].

The strong homeostatic drive to maintain a higher weight and to gain lost weight is meticulously elucidated [19]. After significant weight loss, highly insulin-sensitive and fuel-exhausted fat cells will secrete low leptin [19, 42, 43], hypothesized to be more pronounced in individuals intermittently maintaining, losing and gaining weight [19]. Changes in neuronal activity in the hypothalamus [44] and hindbrain [45–47] in response to a prolonged energy deficit can trigger appetite, reduce satiety and promote positive energy balance [19]. Additional mechanisms, like changes in gut signals and enhanced metabolic regulation, can foster positive energy balance during or after weight loss [19].

In conclusion, individuals with a previous history of weight loss attempts may carry forward the metabolic debt, making it difficult to lose weight with a mild calorie deficit from their calculated daily energy expenditure.

II. Medical

There are many medical reasons for not losing weight. For medical reasons, authors have examined large-effect factors such as hypothyroidism and underactive thyroid, polycystic ovarian syndrome, contraceptive use, medications, surgery and vitamin D deficiency.

1. Hypothyroidism and underactive thyroid

Hypothyroidism, characterized by thyroid hormone deficiency [48], particularly triiodothyronine, influences metabolism. Despite unclear consensus on hypothyroidism directly causing obesity due to limited evidence [49], experiments show treatment effects on weight loss or related markers. A study revealed a 17% reduction in resting energy expenditure with a serum TSH increase (0.1–10 mU/L) from levothyroxine medication [50], hindering weight loss attempts and promoting weight gain. Another study reported hypothyroid patients lost 2.25 ± 2.01 kg of body weight within 12 months after achieving normal thyroid levels through medical treatment [51]. Although seemingly small, combining this effect with lifestyle measures may enhance outcomes.

Observational evidence suggests a positive association between body weight and thyroid-stimulating hormone levels within the normal range [52], but evidence is inconsistent [53]. Obesity may raise thyroid-stimulating hormone levels (subclinical hypothyroidism), contributing to these observations [54]. Maintaining euthyroid status during weight loss interventions helps mitigate hypothyroidism's adverse impact, supporting effective weight loss.

In conclusion, managing euthyroid status is crucial during weight loss interventions to counteract hypothyroidism's hindrance to weight loss efforts.

2. Polycystic ovarian syndrome

Weight gain and obesity can contribute to PCOS/PCOD development in genetically predisposed individuals. Psychological factors, including depression and low self-control, may hinder weight loss efforts in some PCOS patients [55, 56]. However, a systematic review of 14 lifestyle intervention studies (933 participants) found no statistically significant difference in weight loss between women with and without PCOS [57]. Another study comparing a very low-calorie diet for 12 weeks showed no significant difference in weight loss between women with and without PCOS [58]. Psychological effects seem more impactful than physiological changes, calling for future adequately powered studies.

3. Contraceptive use

Recent evidence suggests no clinically and statistically significant short or long-term effects of oral contraceptives on weight in women with normal or higher BMI [59]. A recent secondary analysis suggested an association between oral contraceptive use and weight gain after weight loss, but the small sample size was a limiting factor in the study [60]. The available pieces of evidence are insufficient to estimate the effect of oral contraception on weight loss outcomes during energy restriction. This warrants future clinical trials for a comprehensive understanding of this variable.

4. Medications

Insulin therapy affects weight loss in diabetic individuals, triggering appetite, causing hypoglycemia and increasing body fat [61]. Beta-blockers have reported long-term weight gain, but their effect on weight loss in response to hypocaloric regimens is unclear [62–64].

Antidepressants have indicated an association with obesity in observational studies [65, 66] and there is some evidence suggesting their role in weight gain [67] but causal links and their effect on weight loss outcomes during energy restriction require more investigation. Similar to antidepressants, antibiotics impact also requires large sample RCT studies in the future.

5. Surgery

Bariatric surgeries and other major surgeries restricting movement, such as knee replacement surgeries, are reviewed. The loss of weight after bariatric surgery might be due to the anatomical exclusion of the foregut which may lead to a hormonal upregulation of pancreatic peptide YY, glucagon-like peptide-1 (GLP-1) and gastric inhibitory polypeptide hormones. These factors will improve satiety and reduce hunger, as well as downregulate ghrelin with a subsequent decrease in food intake [68]. As time proceeds, there will be changes in the levels of ghrelin, leptin and incretins diminish, resulting in weight regain as well as insufficient weight loss [69–71]. Additionally, after surgery in most cases, physical inactivity and water retention can lead to weight loss failures.

Elderly patients will not lose as much weight as younger patients due to aging [72]. Also, behavioral and lifestyle changes are found to be crucial for long-term success even after bariatric surgery [73–75].

Studies on other surgeries, like knee replacement surgeries that limit movement, were found to track weight gain, while the weight loss intervention results are very limited in this field. Investigating the impact of common surgeries on energy status is necessary, considering surgical history in weight loss intervention plans.

6. Vitamin D deficiency

Studies indicate a negative correlation between higher BMI/body fat and lower vitamin D concentration [76, 77]. A placebo trial among overweight or obese women with vitamin D deficiency showed no significant difference in weight loss between those receiving oral vitamin D3 and the placebo [78]. These effects were also consistent with one previous similar trial [79]. Vitamin D deficiency does not appear to obstruct meaningful weight loss during lifestyle intervention.

III. Interventional factors

In this section, various interventional factors affecting weight loss results are discussed. Of these, ‘Adherence’ and its impact are quite significant. Therefore, dietary adherence and its associated factors such as ‘Intensity of Energy Deficit’ and ‘Short-Term Fluctuations’ were also reviewed. The other factors covered include ‘Macronutrient-based Diets, ‘Confidence and Bias’ and ‘Perceived Discrimination.’

It is important to mention that the impact of various physical activities and interventions is not in the scope of this study. Hence, an important phenomenon of muscle gain and fat loss that depends on resistance training, which may result in a negligent change in overall weight, is not part of this review study.

1. Dietary adherence

In lifestyle intervention studies, ‘adherence’ is an individual’s ability to stick to dietary or exercise recommendations [80]. A year-long study on four popular diets on weight loss found a significant association (*r* = 0.60; *p* < 0.001) between self-reported adherence and weight loss, but the same was not true for diet type and weight loss (*r* = 0.07; *p* = 0.40) [81]. Similar observations occurred in another study assessing weight loss and dietary adherence across three popular diets [82]. Regardless of the type of diet followed, one-year weight loss was greater in most adherent individuals. Moreover, previous dietary adherence levels are good predictors of future long-term weight regain [80]. In a study on premenopausal women, subjects who showed higher adherence during past low-calorie diet intervention regained 49.9 ± 8.8% of lost weight in two years follow-up, while the lower adherence tertile showed 96.8 ± 12.8% gain [83].

However, adhering to any lifestyle intervention poses a significant challenge [84, 85]. For example, participants in a study by Del Corral et al*. *[83] showed higher adherence to a low-calorie diet but still regained half of their weight after 2 years. Dansinger's study reported overall low adherence across all four intervention diets, with adherence declining over time [81]. A meta-analysis of 27 studies reported only a 60.5% adherence rate [85]. Lack of adherence also leads to large attrition rates in weight loss studies, often underreported [86, 87]. Furthermore, the rate of adherence scores and attrition rates show large variability, i.e., from 28 to 40 percent throughout the literature [87, 88].

Adherence to intervention is positively associated with factors such as greater weight loss [81, 89], reduction of risk parameters [81], self-monitoring [90] and social support [91]. Conversely, it is negatively associated with factors like lack of knowledge, insufficient education about diet, inability to afford healthy food, limited awareness of the benefits of dietary recommendations [92], stress, unrealistic weight loss expectations, poor self-confidence and reduced motivation [86]. Though several associations are identified, a recent Cochrane review highlighted that the available evidence is inadequate to produce optimal guidelines to improve dietary adherence in clinical practice [93].

Initial weight loss success increases motivation and confidence, which as we have reviewed above, improves adherence and reduces chances of weight loss failure. On the other hand, fluctuating results give mixed signals on the success of strategies and can disrupt adherence. Both these factors are studied here about adherence and are identified to be important as either a reinforcer or demotivator for continuing weight loss efforts. Future studies should also use keywords such as ‘not losing’ weight to record findings.

- Intensity of energy deficit

Initial weight loss directly correlates with the intensity of the energy deficit, with both intense and sustainable energy interventions studied extensively. While there's no specific definition for 'low energy/calorie diets,' intense interventions prescribe ~ 800 kcal and a sustained 500–750 kcal deficit model is widely studied and prescribed [94, 95]. Conflicting conclusions exist about the most appropriate energy deficit intensity for weight loss, but both emphasize a positive correlation between initial weight loss and adherence to the intervention [96]. The 500–750 kcal deficit model is relatively easier to follow and may enhance adherence. Setting the right intensity requires discussion with the patient, considering individual motivation and preference, as a mismatch can lead to loss of motivation, and non-adherence, and hinder weight loss efforts.

b. Short-term fluctuations

Variations in glycogen levels [97], constipation and errors in weight or fat loss measuring techniques can cause short-term fluctuations or mistakes in recording progress. A lack of improvements may demotivate individuals and compromise adherence [81, 98]. Educating the patients/clients about these is important to increase awareness and avoid derailing weight loss progress.

2. Macronutrient-based diets

The weight loss benefits of dietary interventions varying in macronutrient compositions are widely studied throughout the literature. In particular, changing protein and carbohydrate compositions in an ad-libitum diet has been shown to have a significant effect on weight loss in several studies [99].

There is some evidence that high-protein diets are beneficial for weight loss [100]. A meta-analysis showed that high-protein diets were modestly beneficial for weight loss compared to standard protein diets (~ 0.8 g/kg) [101]. In the longer term in weight loss interventions, high-protein diets tend to prevent muscle loss, which in turn can positively affect total energy expenditure [100, 101]. Protein also tends to have a higher thermic effect which may partly contribute to additional energy expenditure [102, 103]. In contrast, a diet significantly lower in protein can affect energy expenditure negatively in the short and the longer term [101]. Through several physiological processes, dietary protein induces higher satiety as well [104].

Ketogenic or lower carbohydrate diets (where the majority of the carbohydrate intake is substituted with fat intake) which are significantly lower in carbohydrates tend to show greater weight loss in a shorter (approximately 24 weeks) duration [105–107]. Initial glycogen depletion, higher satiety, appetite suppression and greater metabolic efficiency are some proposed mechanisms through which such low carbohydrate diets might be proven successful [108]. However, if matched for calories, lower carbohydrate diets do not seem to be superior for fat loss compared to higher carbohydrate diets [109].

Hence, concerning carbohydrates, in the long-term, their distribution in the diet is not significant regarding hindrance in weight loss. On the other hand, those on low protein diets may suffer from lean mass loss which will affect the resting metabolic rate in the long-term weight loss interventions. Lean mass maintenance also depends on regularly engaging in resistance training, the discussion of which is beyond the scope of this review. It can be argued that as long as a diet meets a calorie deficit requirement and is sufficiently satiating along with supporting lifestyle interventions such as suitable activity to prevent lean mass loss, the variation in the macronutrient composition of the diet may not play a significant role as the reason for someone not being able to lose weight [110].

3. Confidence and bias

Personal beliefs of the coach, practitioner or researcher about nutrition or exercise intervention may affect the response to behavioral or dietary intervention, and hence, it can directly affect adherence levels in subjects [111]. This can influence the weight loss experienced.

4. Perceived discrimination

Individuals who feel judged by primary care providers for their obesity achieve less weight loss compared to those who do not perceive judgment [112]. Individuals who experienced more obesity stigma reported having less health utility [113]. Obesity care providers can improve their interpersonal communication and apply sensitivity in discussions [112].

IV. Lifestyle factors

Lifestyle factors evaluated that may hinder weight loss include ‘Sleep Deprivation,’ ‘Circadian Misalignment,’ ‘Hydration,’ ‘Alcohol,’ ‘Psychological Factors’ and ‘Consumption of Ultra Processed Foods.’

1. Sleep deprivation

In an experimental setting, a two-week moderate energy restriction study showed that the group sleeping for 8.5 h lost more fat compared to the 5.5-h sleep group (1.4 vs. 0.6 kg, *p* = 0.043) [113]. Despite equal weight loss, the sleep-deprived group lost more muscle mass, indicating the importance of sufficient sleep during a weight loss journey. Sleep-deprived individuals exhibit hormonal changes promoting a protective effect on body fat [116] with greater reductions in resting metabolic rate and increased hunger, possibly due to elevated ghrelin levels (hunger hormone) [115] and a shift in respiratory quotient [114]. Poor sleep quality is also linked to reduced muscle mass and, with even a small reduction in sleep (1.5 h over three weeks), reduced insulin sensitivity and increased weight gain [117, 118]. Overall, the hormonal changes induced by sleep deprivation can make sustaining a restricted-energy diet challenging, leading to increased hunger levels and potentially hindering weight loss efforts.

2. Circadian misalignment—sleep and meals

Circadian rhythms, regulated by a central clock and peripheral clocks, influence various physiological processes [119] through changes in several environmental stimuli such as light, and meals that can alter set rhythms of rest and wakefulness[120, 121]. Following conflicting daily patterns such as shift work creates circadian misalignment, evaluated by observing cortisol and melatonin profiles [122]. This can also reduce total daily energy expenditure [6, 123], and decrease glucose tolerance and leptin levels [124], making weight loss challenging.

Moreover, delaying the main meal deterred the pace and the total amount of weight lost [125]. In another study, women eating more calories at breakfast (50%) than at dinner (14%) lost more weight (5.1 kg difference) and reported better satiety than the other group following opposite meal patterns [126].

Current evidence strongly supports the fact that erratic sleep and eating patterns can cause circadian misalignments and cause a change in energy expenditure due to changes in physical activity, hunger levels and insulin resistance. This can negatively affect weight loss targets during an energy deficit.

3. Hydration

A study from the National Health and Nutrition Examination Survey (NHANES) reported a significant association between elevated body mass index and inadequate hydration after adjusting for confounders [127]. While increased water intake benefits groups aiming to lose weight by improving satiety and reducing energy intake [128–130], dehydration itself is not directly linked to weight loss hindrance in research on humans. Choosing water over calorie-rich drinks can reduce overall energy intake (up to 200 kcal), contributing to weight loss efforts [131].

4. Alcohol

Alcohol's contribution to energy balance, beyond its caloric content, involves inhibiting lipolysis, lipid oxidation and encouraging de-novo lipogenesis [132]. Excessive alcohol consumption can lead to impulsive behavior, unplanned eating events and reduced energy debt [133]. Long-term alcohol consumption, even at low to moderate doses, is associated with insomnia [134], reducing energy expenditure due to sleep deprivation. While studies report minor differences in weight loss related to alcohol consumption, probably from compensatory behavior, the combined effects of inadvertent eating, sleep disruption and reduced fat oxidation hinder long-term weight loss progress [135–137].

5. Psychological factors

Certain psychological conditions, like Binge Eating (BE), are strongly linked to overweight and obesity [138], but when it comes to weight loss, the results are mixed [139–142]. Ongoing BE, more than pre-existing BE, impedes weight loss efforts over four years [143].

Additionally, psychological factors, such as lack of willpower, self-sabotage, self-perception of body image and past stigmatizing experiences related to excess weight, emerge as barriers to weight loss and its maintenance in the long term [144, 145]. Clinical depression and other emotional challenges may also lead to sedentary behavior, overeating [146] and difficulty in adherence to weight loss interventions.

6. Ultra processed foods

A trial comparing ultra-processed and unprocessed diets (as described by the NOVA classification) [147] demonstrated that ultra-processed diets increased energy intake (~ 500 kcal/day) and correlated strongly with weight changes [148]. The inclusion of ultra-processed foods can drastically impact weight loss outcomes, particularly regarding sugar and fiber content.

Free sugars definition excludes lactose, and sugar present in the cellular structures of foods such as fruits and vegetables [149]. The relation of these non-milk extrinsic sugars to obesity or weight gain is not direct [5, 150]. However, a meta-analysis showed that the calories consumed from sugars are inadequately compensated which sheds clear light on the presence of a strong relationship between excessive sugar intake and weight gain [151]. This can likely be due to the response from the reward system that our bodies have developed to foods such as sugars [152] due to lesser satiety of sugary drinks [153] or both.

Based on the same perception, low energy density foods like fiber improve satiety with have increased gastric retention [154]. However, the evidence behind the direct causative effect is equivocal [155] and this might be due to variations in the satiety properties of different types of fibers. The different types of dietary fibers have different physical structures, hydration properties, fermentability and viscosity [156]. Recent studies [157, 158] corroborate this and have shown that some dietary fiber types might be better than others at inducing satiety. Research studies should be cognizant of the types of fibers when including them in weight loss study formulations.

V. Environmental factors

The environmental factors of not losing weight reviewed in the article are ‘Stress,’ ‘Social Support,’ ‘Weather,’ ‘Pollutants’ and ‘Plastics.’ These are external causes that may have an association with not losing weight.

1. Stress

Several studies report an association between psychological stress (job-related or otherwise) and poor emotional health to weight gain [159–161]. However, stress does not always lead to weight gain in all groups [162, 163]. This can be because there is a difference in individual responses to stress. When stress is reciprocated by comfort eating and sleep deprivation, such behaviors can contribute to increased energy intake [164–166]. Additionally, stress can hamper weight loss efforts [167], and can increase the probability of individuals dropping out [166]. A trial comparing groups receiving standard dietary and physical activity instructions that either followed stress management programs or had no such intervention. Stress management program participants lost more weight compared to the control group (1.36 kg/m^2^ difference in body mass index) [168].

As discussed, stress may lead to slower weight loss if it promotes more energy intake. Future studies should shed light on the relationship between different types and levels of stress and weight loss in populations intending to lose weight.

2. Social support

Still, many individuals do not know the role of social support in losing weight. Studies conducted by reported that in-person or internet-based community support can also aid in weight loss [169, 170]. In a weight loss intervention study, a greater number of participants who were recruited along with friends completed the treatment compared to those who participated alone [169]. The prior group even showed better weight loss maintenance. In a survey including the internet weight loss community, themes such as encouragement and motivation, sharing of experience and information, recognition of success and friendly competition were valued and reported to be beneficial by the members [170].

3. Weather

As shown in several studies [171–174], extreme temperatures and ongoing precipitation may lead to a reduction in physical activity and slower weight loss. In such conditions, indoor alternate activities or compensatory dietary behaviors can be considered.

4. Pollutants

Short or long-term exposure to air pollution (a mixture of gases and suspended solid and liquid particles known as particulate matter) leads to increased cardiovascular risk [175]. Promotion of plaque formation, enhanced thrombosis and greater systemic inflammation are probable pathways. This association is more prominent in old age, obesity and chronic disorders [176, 177]. There is some evidence to suggest a negative association between air pollution and intentional weight loss [178] and weight loss after bariatric surgery [179]. However, more studies are needed to investigate the plausible causal relation and the extent of the effect on energy balance and weight.

5. Plastics

Plastics comprise additives such as bisphenol-A (BPA) and nonylphenol, which are associated with body weight imbalance [180]. An in vitro study performed by [181] has shown that these molecules encourage lipid accumulation and promote preadipocyte maturation. A cross-sectional study reported a positive association between BPA and diabetes [182]. Recent reviews done by [183, 184] summarized that several chemicals (obesogens), including those in plastics, can interfere with weight in multiple ways including promoting adipocyte number and size, interfering with hunger and satiety hormones, triggering insulin resistance and decreasing the resting metabolic rate. With the current mechanistic understanding of obesity pathogenesis, limiting exposure to such obesogens can be advantageous in energy deficit.

## Summary of selected studies

The studies selected for the present review are based on the factors responsible for not losing weight. Most of these factors are not interventions. Hence, the no. of not losing cases is not mentioned in the selected review articles. Table 1 shows the details of selected studies.

**Table 1:** Details of selected studies

S. no.	Factors	Authors
I	Biological	
1	Genetics	Ziegler et al. [24] Nikpay et al. [25] Dent et al. [26] Reinhardt et al. [27] Krakoff et al. [28]
2	Anthropometry	Westerterp [29] Heymsfield et al. [30] Ten Hoor et al. [31] Tryon et al. [32] Bautista-Castano et al. [33] Dishman and Gettman [34] Epstein et al. [35] King et al. [36] Kriska et al. [37] Lenoir et al. [21]
3	Metabolic	Dulloo and Schutz [38] Müller et al. [39] Martins et al. [40] Hall et al. [41] Björntorp et al. [42] Löfgren et al. [43] Maclean et al. [19] Bi et al. [44] Chandler et al. [45] Morton et al. [46] Rosenbaum et al. [47]
II	Medical	
1	Hypothyroidism and underactive thyroid	Chaker et al. [48] Garber et al. [49] Al-Adsani et al. [50] Ríos-Prego et al. [51] Fox et al. [52] Manji et al. [53] Song et al. [54]
2	Polycystic ovarian syndrome	Barber et al. [55] Lim et al. [56] Kataoka et al. [57] Nikokavoura et al. [58]
3	Contraceptive use	Mayeda et al. [59] Caldwell et al. [60]
4	Medications	Russell-Jones and Khan [61] Shi et al. [65] Gafoor et al. [66] Olguner et al. [67] Gammone et al. [64] Lamont et al. [62] Lee et al. [63]
5	Surgery	Jirapinyo et al. [68] Bohdjalian et al. [69] Santo et al. [70] Zalesin et al. [71] Gualano et al. [74]
6	Vitamin D	Arunabh et al. [76] Parikh et al. [77] Mason et al. [78] Zittermann et al. [79]
III	Interventional factors	
1	Dietary adherence	Gibson and Sainsbury [80] Dansinger et al. [81] Alhassan et al. [82] Del Corral et al. [83] World Health Organization [84] Rogers et al. [85] Grave et al. [86] Dombrowski et al. [87] Franz et al. [88] Del Corral et al. [89] Klein et al. [90] Burke et al. [91] Ayele et al. [92] Desroches et al. [93]
a	Intensity of Energy Deficit	Koliaki et al. [94] Parretti et al. [95] Greenberg et al. [96]
b	Short-term fluctuations	Kreitzman et al. [97] Hall et al. [148] Dansinger et al. [81]
2	Macronutrient-based diets	Bellissimo and Akhavan [99] Moon and Koh. [100] Wycherley et al. [101] Halton and Hu [102] Westerterp-Plantenga et al. [103] van der Klaauw et al. [104] Dashti et al. [105] Hussain et al. [106] Sato et al. [107] Paoli [108] Leidy et al. [110] Hall et al. [109]
3	Confidence and bias	Rollwage et al. [111]
4	Perceived discrimination	Gudzune et al. [112] Wee et al. [113]
IV	Lifestyle factors	
1	Sleep deprivation	Wee et al. [113] Nedeltcheva et al. [114] Spiegel et al. [115] Klok et al. [116] Buchmann et al. [117] Robertson et al. [118]
2	Circadian misalignment—sleep and meals	Johnston [119] Oda [120] Ruddick-Collins et al. [121] Johnston et al. [122] Buxton et al. [123] Scheer et al. [124] Garaulet et al. [125] Jakubowicz et al. [126]
3	Hydration	Chang et al. [127] Muckelbauer et al. [128] Dennis et al. [130] Daniels and Popkin [129] Popkin et al. [131]
4	Alcohol	Siler et al. [132] Kase et al. [133] Stein and Friedmann, [134] Chao et al. [135] French et al. [136] Levinson and Rodebaugh [137]
5	Psychological factors	Poraj-Weder et al. [138] Blaine and Rodman [139] Masheb et al. [140] [141] Sherwood et al. [142] Chao et al. [143] Herriot et al. [144] Garip, and Yardley [145] Raman et al. [146]
6	Ultra processed foods	Monteiro et al. [147] Hall et al. [148] Swan et al. [149] Blundell et al. [150] Scientific Advisory Committee on Nutrition [151] Wiss et al. [152] Shearrer et al. [153] Van Itallie [154] Wanders et al. [155] Hervik and Svihus [156] Rebello et al. [157] Salleh et al. [158]
V	Environmental factors	
1	Stress	Block et al. [159] Tucker and Earl [160] Cotter and Kelly [161] Kivimäki et al. [162] Fowler-Brown et al. [163] Sims et al. [164] Scott et al. [165] Geiker et al. [166] Pellegrini et al. [167] Xenaki et al. [168]
2	Social support	Wing and Jeffery [169] Hwang et al. [170]
3	Weather	Baranowski et al. [171] Tucker and Gilliland [172] Klenk et al. [173] O’Neill et al. [174]
4	Pollutants	Brook et al. [175] Dubowsky et al. [176] Li et al. [177] Ustulin et al. [178] Ghosh et al. [179]
5	Plastics	Diamanti-Kandarakis et al. [180] Wada et al. [181] Lang et al. [182] Darbre [183] Shahnazaryan et al. [184]

## Strength and limitations of this article

### Strength

- Numerous review papers and randomized trials try to uncover the best way to lose weight. This scoping review adds to the limited reviews looking at why someone may not be able to lose weight despite participating in an intervention.
- This is an extensive review with multiple factors associated with not losing weight highlighted

### Limitations

- Factors associated with not losing weight that the authors categorized in this article are purely based on the authors’ research and/or client-handling background. Hence, there could be chances of bias.
- There is no years restricted for selecting the review article
- Factors such as gut health and muscle gain were excluded owing to the length of the study.
- The impact of physical activity has not been covered in this review and should be considered in future studies.
- Fluctuations in the muscle mass were not covered due to the length of the article.

### Future research

- Whenever a randomized clinical trial is conducted along with successful weight loss results, reporting should be done on ‘not-losing’ cases as well.
  - Need for monitoring the body composition parameters apart from weight loss alone
  - Consider fat loss and muscle gain in the case of not losing weight.

## Suggestions for addressing not losing weight

Based on the review on factors of not losing weight, a few suggestions for clinical settings are given below:

### Accepting interindividual variability

This study is a modest attempt to highlight that energy requirements for individuals vary widely not only due to poor dietary adherence but also due to the interindividual biological variation in energy expenditure responses to energy deficit [4]. Furthermore, an individual attempting weight loss should consider their biological and medical background, environmental impact, lifestyle factors and intervention factors. Although these factors may pose challenges for some, a slow pace of weight loss should not deter individuals from their weight loss journey. Medical practitioners or weight loss experts can make them aware of these factors, emphasizing the uniqueness of their journey and the importance of focusing on a long-term, sustained weight loss strategy.

### Adherence

Many individuals seeking weight loss often search for a perfect diet or weight loss method. This review highlights that adherence to a calorie-restricted diet over the long term will invariably give the desired results. It is prudent to then consider the sustainability of maintaining a calorie-restricted diet, over struggling with restricting certain macronutrients or meal frequency, etc.

### Contextualizing short-term fluctuations

Weight loss is never linear and the fluctuations in the amount of weight lost week on week are not consistent. The efforts to lose weight need to be consistent despite the scale not moving due to short-term factors such as water retention.

### Considering environmental factors

Stress affects the body in many detrimental ways, one of which is making it difficult to lose weight. One should practice stress reduction techniques to ensure that their impact is minimized during weight loss interventions. Having a support group, maintaining physical activity despite the weather conditions and reducing exposure to pollutants and plastics will go a long way toward more positive results from weight loss interventions.

## Conclusion

In dietary interventions, the interindividual variability in weight loss can be explained by several factors that may inhibit weight loss by undermining the intended energy deficit. In clinical practice, the role of such variables should be considered while planning personalized interventions targeting optimal weight loss. It is also crucial to understand that weight loss is a product of overall lifestyle and not a few individual elements in isolation. In future research, relevant factors should be considered while designing and analyzing studies targeting weight loss. Such interpretations can guide practitioners to incorporate evidence more effectively in their clinical practice.

This review identifies and clarifies the role of several factors that may hinder weight loss after the exploration of existing evidence by the authors. However, this review has not covered all such factors. Studies with insignificant outcomes are often not published, and this review is limited to published literature. Additionally, the authors selected studies that they thought were relevant for clinical applications. Moreover, it is difficult to estimate a cause-and-effect relationship in many of the discussed studies. Thus, it can be concluded that the effectiveness of respective interventions by simply observing the ‘general behavior of the groups’ is not always applicable in clinical practice. Each patient must be monitored and advised as per individual requirements and challenges.

## Data Availability

All data gathered during this study are included in the published article.

## Abbreviations

BE: Binge Eating
BMI: Body mass index
FTO: Fat mass and obesity-associated gene
MC4R: Melanocortin-4 receptor
MTIF3: Mitochondrial translational initiation factor 3
PCOD: Polycystic ovarian disease
PCOS: Polycystic ovarian syndrome
PPARG: Peroxisome proliferator-activated receptor gamma
RCTs: Randomized controlled trials
SCN: Suprachiasmatic nucleus
TSH: Thyroid stimulating hormone
